# Structural insights reveal the specific recognition of roX RNA by the dsRNA-binding domains of the RNA helicase MLE and its indispensable role in dosage compensation in *Drosophila*

**DOI:** 10.1093/nar/gky1308

**Published:** 2019-01-15

**Authors:** Mengqi Lv, Yixiang Yao, Fudong Li, Ling Xu, Lingna Yang, Qingguo Gong, Yong-Zhen Xu, Yunyu Shi, Yu-Jie Fan, Yajun Tang

**Affiliations:** 1 Hefei National Laboratory for Physical Sciences at Microscale and School of Life Sciences, University of Science and Technology of China, Hefei, Anhui 230026, China; 2 State Key Laboratory of Virology, Hubei Key Laboratory of Cell Homeostasis, College of Life Sciences, Wuhan University, Wuhan 430072, China; 3 CAS Center for Excellence in Biomacromolecules, Institute of Biophysics, Chinese Academy of Sciences, Beijing 100101, China; 4 Key Laboratory of Insect Developmental and Evolutionary Biology, Institute of Plant Physiology and Ecology, Shanghai Institutes for Biological Sciences, Chinese Academy of Sciences, Shanghai 200032, China

## Abstract

In *Drosophila*, dosage compensation globally upregulates the expression of genes located on male single X-chromosome. Maleless (MLE) helicase plays an essential role to incorporate the roX lncRNA into the dosage compensation complex (MSL-DCC), and such function is essentially dependent on its dsRNA-binding domains (dsRBDs). Here, we report a 2.90Å crystal structure of tandem dsRBDs of MLE in complex with a 55mer stem-loop of roX2 (R2H1). MLE dsRBDs bind to R2H1 cooperatively and interact with two successive minor grooves and a major groove of R2H1, respectively. The recognition of R2H1 by MLE dsRBDs involves both shape- and sequence-specificity. Moreover, dsRBD_2_ displays a stronger RNA affinity than dsRBD_1_, and mutations of key residues in either MLE dsRBD remarkably reduce their affinities for roX2 both *in vitro* and *in vivo*. In *Drosophila*, the structure-based *mle* mutations generated using the CRISPR/Cas9 system, are partially male-lethal and indicate the inter-regulation among the components of the MSL-DCC at multiple levels. Hence, our research provides structural insights into the interactions between MLE dsRBDs and R2H1 and facilitates a deeper understanding of the mechanism by which MLE tandem dsRBDs play an indispensable role in specific recognition of roX and the assembly of the MSL-DCC in *Drosophila* dosage compensation.

## INTRODUCTION

X-chromosomal dosage compensation processes exist in a wide range of eukaryotic organisms (1). This biological process is essential for balancing the expression levels of X-linked genes caused by the unequal number of X chromosomes between males and females (2). Different species have evolved different strategies. In female mammals, one of the two X chromosomes is transcriptionally inactivated, through a process called X chromosome inactivation (3,4). In contrast to mammals, the compensation process in male *Drosophila* is mediated by the double upregulation of the transcription of genes located on the single X chromosome in males (5).

Dosage compensation in *Drosophila* is achieved by a ribonucleoprotein complex MSL-DCC (6). The complex consists of five proteins (MSL1, MSL2, MSL3, males absent-on-the-first (MOF) and MLE) together with at least one of the two long non-coding RNAs, called RNA on the X 1 and 2 (roX1 and roX2) (7,8). The MSL-DCC selectively binds to the discontinuous high-affinity sites (HASs) of the male X chromosome and X chromosome-widely acetylates histone H4 lysine 16 (H4K16Ac) (9,10). The acetylation mediated by the acetyltransferase activity of MOF loosens the chromatin fiber, promotes active gene transcription and upregulates mRNA levels (11,12). The transcriptional levels of male X chromosome-linked genes are globally upregulated by ∼2-fold, compensating for the lack of one X chromosome in males (13).

Following unwinding and remodeling by MLE, roX RNA exposes binding sites for MSL2 and triggers the assembly of the MSL-DCC (12,14). MSL2 is also essential for roX RNA incorporation in the MSL-DCC and cooperates with MLE to spread to the HASs on the X chromosome (15,16). According to the results of a protease hydrolysis assay, MLE only interacts with the roX RNAs and has little contact with other components of the MSL-DCC (16). In absence of MLE helicase, roX RNA fails to be incorporated into the MSL-DCC (17). Thus, the mechanism of MLE helicase-mediated unwinding and remodeling of roX RNA is the first step in dosage compensation in *Drosophila*.

**Figure 1.**
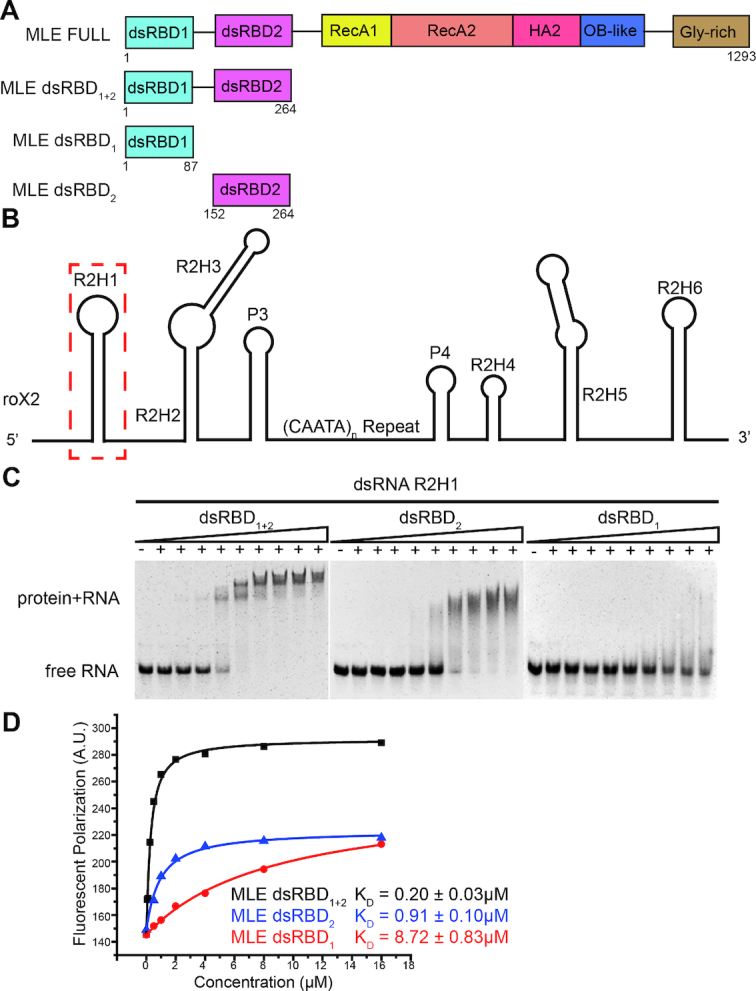
The MLE dsRNA binding domains bind the first stem-loop of roX2–R2H1. (**A**) The domain organization of MLE helicase; the constructs used in this study are indicated. (**B**) Structural model of roX2 exon-3 showing the eight stem-loop structural domains linked together by flexible single-stranded linkers. The first stem-loop of roX2–R2H1 is highlighted with a dashed red box. (**C**) EMSAs with unlabeled 55mer R2H1 and identical increasing concentrations of MLE dsRBD constructs. (**D**) The binding affinities of 3′-FAM-labeled 55mer R2H1 for dsRBD_1_ (red), dsRBD_2_ (blue), dsRBD_1+2_ (black) determined by FP experiments are shown.

The lengths and sequences of roX1 and roX2 are quite different but they both contain conserved uridine-rich regions known as the roX boxes (18,19). As reported, MLE helicase exhibits specificity for uridine nucleotides, rationalizing the conservation of uridine-rich sequences in roX RNAs (20). Selective 2′-hydroxyl acylation analyzed by primer extension and parallel analysis of RNA structure confirmed that roX2 exon-3 consists of eight stem-loops connected by flexible single-stranded linkers (Figure 1B) (19). RoX2 is split into two clusters: four stem-loops at the 5′ end that interact with MLE helicase in an adenosine triphoshate (ATP)-independent manner (R2H1, R2H2, R2H3 and P3) and the four stem-loops at the 3′ end that interact in an ATP-dependent manner (P4, R2H4, R2H5 and R2H6), with CAATA repeats connecting the two clusters. The ATP-independent interaction with roX2 is mediated by the N-terminal tandem dsRBDs of MLE (19). Moreover, among the 5′ cluster of roX2, the first helical structure of roX2 (R2H1) is the most important for MLE dsRBD binding (19,21).

MLE is an ATP-dependent DEXH box RNA/DNA helicase (22,23). Generally, MLE helicase unwinds blunt-ended dsRNA or RNA/DNA hybrid duplexes in the 3′ to 5′ direction (20). Similar to other RNA helicases, MLE contains two conserved RecA domains that are responsible for the RNA-dependent ATPase and ATP-dependent RNA unwinding activities (20,24). MLE also contains auxiliary domains, including two tandem dsRBDs at the N-terminus and a helicase-associated 2 (HA2) domain, an OB-fold domain and a glycine-rich region at its C-terminus (20). Many RNA helicases use RecA domains to remodel RNA while employing auxiliary domains to bind the substrate, interact with protein partners and regulate the catalytic activity (20,25). In the complex structure of MLE_core_–U_10_–ADP-AlF_4_ (PDB ID: 5AOR), auxiliary domains of MLE_core_ coordinate with RecA domains to specifically recognize the UxUUU motif (20). The structure of MLE_core_–U_10_–ADP–AlF_4_ complex has provided insights into the mechanisms of ATP-dependent MLE helicase activity and the ssRNA binding status (20). The ATP-independent interaction between MLE and roX2 is mainly mediated by the N-terminal tandem dsRBDs of MLE (19). However, the mechanisms revealing how MLE dsRBDs specifically targets the roX2 dsRNA and the interaction mediate the dsRNA unwinding remain largely unknown.

DsRBDs are well-characterized dsRNA binding domains of ∼65–75 amino acids that adopt a conserved αβββα fold (26). Proteins that contain tandem dsRBDs interact with dsRNA through more than one dsRBD and participate in many biological processes, such as RNA silencing, RNA editing, RNA processing, etc. (27–29). Human RNA helicase A (RHA, also named as DHX9) utilizes its N-terminal tandem dsRBDs for siRNA recognition to promote the formation of the active RISC (RNA-induced silencing complex) (30). The two dsRBDs of rat ADAR2 (adenosine deaminases that act on RNA) specifically recognize the R/G site of GluR-B to modify adenosines to inosines within RNA transcripts for recoding genomic information (31). The structures of many complexes have been reported, but limited descriptions of the structures of dsRBDs with their natural RNA target are available. Regarding the N-terminal tandem dsRBDs of MLE, dsRBD_2_ displays a stronger binding capacity for roX RNA than dsRBD_1_ (32). DsRBD_2_ is required for the ATPase and helicase activities of MLE, whereas deletion of dsRBD_1_ does not influence the helicase activity of MLE (23,32). However, both of domains are indispensable for male X chromosome targeting (23,32).

To obtain structural insights into roX2 recognition and MSL-DCC assembly facilitated by MLE dsRBDs, we determine the crystal structure of the tandem dsRBDs of MLE in complex with R2H1. We show that MLE tandem dsRBDs interact with R2H1 by cooperatively binding either side of the dsRNA. Through the structural analyses, we identify the key residues of MLE dsRBDs that are responsible for the specific recognition of R2H1. Structure-based mutations in MLE dsRBDs significantly reduce the roX2 binding affinity of MLE, result in male lethality, and disrupt the accumulation of the MSL-DCC on X-chromosome in male flies. Intriguingly, the mutant of the key residues in the roX2 binding of MLE dsRBD_2_ not only causes comparable male mortality as the deletion of the tandem dsRBDs, but also makes MLE completely lose the ability of X-chromosome-specific localization. Hence, using a combination of biochemical, structural, and functional studies, we provide a detailed description of the specific recognition of a natural dsRNA by MLE dsRBDs that facilitates a deeper understanding of the mechanism by which MLE utilizes the tandem dsRBDs to recognize roX RNA and elaborately regulate the assembly of the MSL-DCC.

## MATERIALS AND METHODS

### Protein expression and purification

The full-length *Drosophila mle* gene was cloned in the pEASY-T vector (TransGen Biotech). Different MLE dsRBDs constructs: dsRBD_1_ (residues 1–87), dsRBD_2_ (residues 152–264) and dsRBD_1+2_ (residues 1–264), were amplified and cloned into a modified pET28a (Novagen) plasmid. The modified pET-28a plasmid contained an N-terminal SUMO-tag and a ULP1 protease cleavage site. The proteins were expressed in *Escherichia coli* BL21 (DE3) cells (Novagen) cultured in LB medium at 37°C to OD_600_ = 1.0, and then shifted to 16°C for 24 h after induction with 0.5 mM isopropyl-β-D-thiogalactopyranoside (IPTG). Bacterial pellets were resuspended in buffer A (20 mM Tris and 1 M NaCl, pH 7.5) and lysed by sonication on ice. The crude lysate was then centrifuged at 14000 rpm for 30 min at 4°C. The supernatant was applied to a Ni-NTA column (QIAGEN), followed by size exclusion chromatography using a Superdex 200 (GE Healthcare) column. After cleavage with ULP1 protease overnight at 16°C to remove the SUMO-tag, an additional Ni-NTA column purification step was employed. The purified protein was concentrated to ∼20 mg/ml in buffer B (50 mM Na_2_HPO_4_, 150 mM NaCl and 5 mM TCEP, pH 7.0) and stored at −80°C. All mutants were generated using a MutanBEST kit (TaKaRa) and confirmed by DNA sequencing. The mutant proteins were purified using the protocol described above.

### RNA preparation

The RNA used for crystallization, electrophoretic mobility shift assays (EMSA) and fluorescent polarization assays (FP) is a 55mer RNA. It is the first stem-loop of roX2 exon-3 at its N-terminus and was transcribed and purified *in vitro*. The DNA template used to transcribe the 55mer RNA was synthesized by TaKaRa. Bio, Inc., and dissolved in diethyl pyrocarbonate (DEPC)-treated water to a final concentration of 100 mM. The reaction mixture comprised 10 mM Tris, 10 mM DTT, 10 mM NTPs, 40 mM MgCl_2_, 0.3 mM T7 template, 0.3 mM DNA templates and 3 mg/ml T7 polymerase. The reaction was performed at 37°C for 4 h. After transcription, the transcription products were treated with 0.1 total volume (0.1 V) of 0.5 M ethylenediaminetetraacetic acid (EDTA), 0.1V of 5 M NaCl and 3V of absolute alcohol and incubated at −40°C overnight. Then, the transcription products were centrifuged, the supernatant was discarded and the precipitated RNA was dissolved in 1.5 ml of DEPC-treated water. An equal volume of RNA loading buffer was added (TaKaRa), incubated at 90°C for 5 min and cooled on ice for 5 min. The RNA samples were separated on 12% denaturing polyacrylamide gel and purified using Elutrap (Whatman). The final 55mer R2H1 was dialyzed against DEPC-treated water, concentrated to 1 mM and stored at −80°C. Prior to use, the RNA substrate was heat-denatured at 95°C for 5 min and annealed on ice for 5 min.

### Protein crystallization, data collection and structure determination

Native and selenomethionine (SeMet)-derivative MLE dsRBD_1+2_ were directly added to the prepared R2H1 at a 1:1.5 molar ratio followed by separation on a Superdex 200 (GE Healthcare) column in buffer C (20 mM Tris and 150 mM NaCl, pH 7.5) to remove the excess RNA. The complex was concentrated to a final concentration of ∼25 mg/ml. Crystals of the complex were grown at 20°C via the hanging drop vapor diffusion method, with the mother liquor containing 200 mM lithium citrate tribasic tetrahydrate and 20% PEG3350. Crystals were soaked in mother liquor supplemented with 20% glycerol before being flash-frozen in liquid nitrogen. X-ray diffraction data for the crystals were collected on beamline 19U of the Shanghai Synchrotron Radiation Facility (SSRF). The data were processed with HKL2000 and programs in the CCP4 suite. Single wavelength anomalous scattering data were collected from a crystal of the SeMet-derivative MLE dsRBD_1+2_–R2H1 complex. The initial phase was calculated using AutoSol in PHENIX, and the initial model was built using AutoBuild in PHENIX (33). The initial model was then completed through several cycles of manual model rebuilding in COOT (34) and refinement in REFMAC5 (35). The structure of the MLE dsRBD_1+2_–R2H1 complex was determined by molecular replacement with the program MOLREP (36) in CCP4i. All initial models were refined using the maximum likelihood method implemented in REFMAC5 (35) as part of CCP4i program suite and rebuilt interactively using the program COOT (34). Final refinement strategies included XYZ coordinates, individual B-factors, occupancies, and automated correction of N/Q/H errors using PHENIX. Crystallographic parameters are listed in Supplementary Table S1. All images of the structures were prepared using PyMOL (http://www.pymol.org/).

### Circular Dichroism measurements (CD)

Far-UV circular dichroism (CD) spectra of the MLE dsRBD_1+2_ and its mutants were determined using an Applied Photophysics Chirascan spectrometer at 298 K. The spectra were recorded at wavelengths ranging from 195 to 260 nm using a 0.05 cm path length cell. The protein samples were diluted to 0.1 mg/ml with CD buffer (50 mM Na_2_HPO_4_ and 150 mM NaCl, pH 7.5). A buffer-only reference was subtracted from each curve. All samples were tested in triplicate.

### Size exclusion multi-angle light scattering (SEC-MALS)

Size exclusion multi-angle light scattering data were collected using an AKTA pure system (GE Healthcare) with a Superdex 200 Increase 10/300 GL column (GE Healthcare) at a flow rate of 0.6 ml/min in buffer D (20 mM Tris, 150 mM NaCl and 2 mM MgCl_2_ at pH 8.0). The system was coupled on-line to an 18-angle MALS detector (DAWN HELEOS II, Wyatt Technology) and a differential refractometer (Optilab T-rEX, Wyatt Technology). Molar mass determination was calculated using ASTRA 7.0.1.24 software.

### Electrophoretic mobility shift assays (EMSA)

All RNA-binding reactions were performed in binding buffer D. Prior to use, the RNA substrate was heat-denatured at 95°C for 5 min and annealed on ice for 5 min. A total of 10 μl of the binding reaction contained 5 μl of 1200 nM unlabeled R2H1 and 5 μl of MLE dsRBDs at various concentrations. MLE dsRBDs were first diluted to 120 μM, followed by successive 2-fold dilutions to a final concentration of 46.87 nM. Reactions were incubated at room temperature for 40 min and resolved on 6% native polyacrylamide gels, unless stated otherwise.

### Fluorescent polarization assays

The lyophilized 3′-FAM (carboxyfluorescein)-labeled RNA oligomer (R2H1) was purchased from TaKaRa Bio, Inc., dissolved in DEPC-treated water to a final concentration of 100 μM and stored at −80°C. The stock (100 μM) was diluted to 80 nM in dilution buffer D. Equilibrium dissociation constants of RNA and different MLE dsRBD constructs were determined by measuring FP, as previously described. MLE dsRBD constructs were first diluted to 20 times the highest concentration used in the binding system, and then successively diluted 2-fold until the lowest desired concentration was reached. Before the assay, 100 μl of 80 nM fluorescence-labeled RNA were mixed with 100 μl of protein stocks from the diluted series and incubated for 15 min. Samples were then excited at 485 nm, and FP was detected at 525 nm using a SpectraMax M5 (Molecular Devices) plate reader at 20°C. All FP data were well fitted to a 1:1 binding model and were expressed as follows:

\begin{equation*}{\rm{FP\ = F}}{{\rm{P}}_{{\rm{ini}}}}{\rm{\ + }}\frac{{{\rm{max}}}}{{{\rm{2nR}}}}{\rm{ \times }}\left( {{{{K}}_{\rm{d}}}{\rm{ + P + nR - }}\frac{{{\rm{max}}}}{{{\rm{2nR}}}}\sqrt {{\rm{ - 4nPR + }}{{\left( {{{{K}}_{\rm{d}}}{\rm{ + P + nR}}} \right)}^{\rm{2}}}} } \right)\end{equation*}

where FP is the observed total polarization, FP_ini_ is the initial FP of RNA without any protein, P is the protein concentration, R is the concentration of labeled RNA, n is the binding stoichiometry (protein: RNA ratio) and *K*_d_ is the equilibrium dissociation constant. Standard errors were obtained by fitting the data to the above equation

### Cell culture and RNA immunoprecipitation (RIP)

To generate GFP-MLE_FL_ expression vectors, cDNA encoding full-length *Drosophila* MLE gene was cloned into GFP-pAc5.1 plasmid, resulting in the expression of MLE_FL_ fused to an N-terminal GFP. All mutants and deletions of MLE_FL_ were generated using a MutanBEST kit (TaKaRa) and confirmed by DNA sequencing. For RIP, GFP-tagged wild-type or mutant MLE_FL_ expression vectors were used to transfect exponentially grown stable S2 cell lines. Non-transfected S2 cells served as the control. For each IP, 1 × 10^8^ S2 cells were collected, washed once with PBS and flash frozen in liquid nitrogen. Then, the cell pellet was thawed on ice and resuspended in buffer E (10 mM Tris, 150 mM NaCl and 0.5 mM EDTA, pH 7.5, in DEPC-treated H_2_O). A Roche complete protease inhibitor cocktail, PMSF (Solarbio) and RNasin (Promega) were then added. The cells were lysed by sonication on ice for 2 min. The lysates were then centrifuged at 14000 rpm for 30 min at 4°C. Input material (10%) was kept for RNA and protein analyses. Each supernatant was incubated with 25 μl of GFP-Trap beads (Chromotek) for 2 h at 4°C on a rotating wheel. The beads were extensively washed and then incubated with Proteinase K (100 μg in buffer E with 0.5% sodium dodecyl sulphate) for 45 min at 55°C. RNA was extracted using phenol:chloroform:isoamylalcohol (125:124:1, pH 5.6) (Solarbio) once and chloroform once and then precipitated with EtOH. RNA samples (Input and IP) were subjected to quantitative reverse-transcription polymerase chain reaction (qRT-PCR) with SYBR green dye (Applied Biosystems) using primers specific for roX2 and Pka (Supplementary Table S2). RNA enrichment of wild-type or mutant MLE_FL_ is calculated as IP/Input and normalized to wild-type MLE_FL_. The Pka RNA served as an MLE-unbound control in each experiment. Input proteins were analyzed by western blotting using GFP (Abcam) and Lamin (DSHB) antibodies, respectively.

### *Drosophila* culture and mle mutagenesis

Culture and crosses of *Drosophila* were conducted on standard medium at 25°C. Three *mle* mutant strains containing precise deletions or substitutions were generated using the CRISPR/Cas9 system (37). For the generation of each allele, the guide RNA-expressing plasmids and the donor plasmid containing the fragment with deletions or substitution as well as ∼1 kb flanking homologous arms were co-injected into embryos of transgenic line *nos-Cas9* (attP2) by UniHuaii Technology Company. Primers located outside the range of homologous arms and allele-specific primers were used for PCR to screen for the desired mutant alleles and PCR products of all the acquired alleles were sequenced for further verification. At last, the *mle* alleles were balanced over CyO GFP to distinguish the homozygotes in early developmental stages.

### Measurement of gene expression

Samples were prepared from four to five biological replicates of second instar male of *mle^ΔRBDs^* larvae and adult male of *mle^subRBD1^* and *mle^subRBD2^* flies. Total RNA was extracted using Trizol reagent (Invitrogen) and reverse transcription was performed using the First Strand cDNA Synthesis Kit (RevertAid, Thermo) and random hexamer primers, following DNase I (TaKaRa) treatment. The cDNA samples were subjected to qPCR with SYBR green dye (Applied Biosystems) using specific primers (genes and primer information are provided in Supplementary Table S2). Each qPCR was repeated at least three times. Expression levels were normalized to the autosomal gene *Pka*. The expression level of each gene in the mutant strain was normalized to the corresponding gene expression level in the *wild-type* stain. Standard deviations within each experiment were calculated.

### Determination of male viability

All of the male and female adults from at least three independent vials were counted daily until the final end of eclosion to determine male viability. The ratio of the number of males/number of females carrying each allele relative to that of *wild-type* was used to represent the male viability. Moreover, the viability of third instar male larvae was assessed under the microscope. PCR amplification of the *ary* gene on the Y chromosome was performed to distinguish the male second instar larvae.

### Immunoblotting and immunostaining of chromosomes

The rabbit antibodies against MLE, MSL2 and MOF used in this study were obtained from Dr Mitzi I. Kuroda. For immunoblotting, thirty third instar male larvae were used for protein extraction to comprise each sample. Routine sodium dodecyl sulphate-polyacrylamide gel electrophoresis and western blotting procedures were performed. Immunostaining was performed exactly according to *Drosophila* Protocols (38). Antibodies against MLE, MSL2 and MOF were used at dilutions of 1:100, 1:50 and 1:50, respectively. The secondary antibody conjugated to Alexa Fluor 594 was obtained from Jackson ImmunoResearch (111585003). Images were captured using a confocal laser scanning microscope (FluoView FV10i, Olympus).

## RESULTS

### The tandem dsRBD domains of MLE bind to R2H1 together

Previous studies have revealed that MLE utilizes its N-terminal tandem dsRBDs to recognize the 5′ hairpin cluster of roX2 (R2H1, R2H2 and R2H3) in an ATP-independent manner—especially the helical region of R2H1 (19,21). Consistent with previous *in vivo* studies, our *in vitro* FP assay revealed that R2H1 is the strongest substrate for the binding of MLE dsRBDs among 5′ hairpin cluster of roX2 *in vitro* (Supplementary Figure S4C). Subsequently, we expressed different MLE dsRBD constructs to evaluate the dsRNA-binding potential of MLE dsRBDs for R2H1 (Figure 1A and Supplementary Figure S1A). Notably, dsRBD_1_ and dsRBD_2_ have a considerably weaker capacity to interact with R2H1 than tandem dsRBD_1+2_ (Figure 1C). However, both individual dsRBDs and tandem dsRBDs were able to shift the R2H1 (Figure 1C). Clearly, MLE dsRBD_2_ shifted the dsRNA in a dispersive interaction, whereas MLE dsRBD_1_ displayed a weak interaction with the substrate (Figure 1C). Moreover, according to the FP assays, MLE dsRBD_1+2_ binds to R2H1 with a *K*_d_ value of 0.20 ± 0.03 μM, whereas individual dsRBD_1_ and dsRBD_2_ bind to R2H1 with an ∼44- and ∼4.5-fold weaker affinity than MLE dsRBD_1+2_, respectively (Figure 1D). Collectively, MLE tandem dsRBDs interact with R2H1 more efficiently than either dsRBD alone, suggesting that dsRBD_1_ and dsRBD_2_ function together.

We crystallized and determined the structure of the complex of MLE dsRBD_1+2_ with the natural substrate–R2H1 to further characterize the interactions between MLE dsRBDs and R2H1. The structure of the complex was subsequently refined to a resolution of 2.90 Å in space group R32. The crystal structure was determined by combining phases from partial molecular replacement using the homologous structures of dsRNA-binding domains of human RNA helicase A-DHX9 (DHX9 dsRBD_1_ PDB ID: 3VYY and DHX9 dsRBD_2_ PDB ID: 3VYX) and single wavelength anomalous scattering data obtained from crystals of the SeMet-derivative complex. At last, the *R*_work_ and *R*_free_ of the structure of the MLE dsRBD_1+2_–R2H1 complex were refined to 19.09 and 24.37%, respectively. The detailed crystallographic statistics are summarized in Table 1.

**Table 1.** Data collection and refinement statistics

Data collection	MLE dsRBD_1+2_–R2H1
Beamline	19U, SSRF
Space group	R32
Wavelength (Å)	0.9792
Resolution (Å)	46.55–2.90 (3.00–2.90)
Cell dimensions	
a, b, c (Å)	108.48 108.48 347.06
α, β, γ (°)	90 90 120
Unique reflections	17898 (1762)
Completeness (%)	99.9 (99.6)
Redundancy	9.4 (9.6)
I/σI	22.5 (2.7)
*R*_merge_ (%)	12.0 (78.7)
**Refinement**	
*R*_work_ (%)	19.09
*R*_free_ (%)	24.37
No. of atoms	3586
No. of protein atoms	2538
No. of nucleic acid atoms	1048
Average B factors (Å^2^)	
Protein	70.37
nucleic acid	85.05
Root mean square deviations	
Bond lengths (Å)	0.009
Bond angles (°)	1.183
Ramachandran plot	
Favored (%)	91.36
Allowed (%)	7.72
Disallowed	0.93

Statistics for the highest-resolution shell are shown in parentheses.

### Overall structure of MLE dsRBD_1+2_ in complex with R2H1

**Figure 2.**
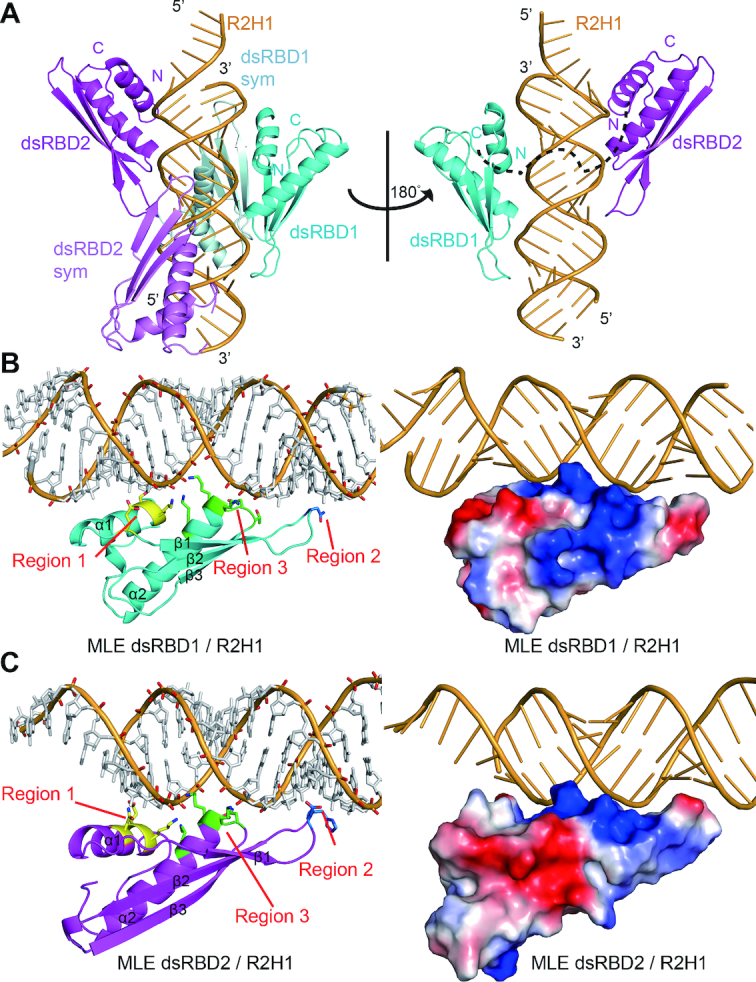
Structural overview of MLE dsRBD_1+2_ in complex with R2H1. (**A**) Cartoon representation of the MLE dsRBD_1+2_–R2H1 complex in two orientations related by a 180° rotation around a vertical axis. Left panel: the asymmetric unit consists of the 55mer R2H1 (bright orange), dsRBD_1_ (cyan) and dsRBD_2_ (magenta). Symmetry equivalent domains are shown in pale cyan and violet, respectively. Right panel: a view of the complex with symmetry equivalent protein domains removed for clarity. The linker between the domains is represented as a black dotted line. (**B**) Overall structure of MLE dsRBD_1_ in complex with R2H1. Left panel: cartoon view of the structure of MLE dsRBD_1_ in complex with R2H1. The critical residues belonging to regions 1, 2 and 3 required for dsRNA recognition are shown in stick mode and are colored in yellow, blue and green, respectively. Right panel: the electrostatic potential of the MLE dsRBD_1_–R2H1 complex is shown, in which positively charged, negatively charged and neutral areas are represented in blue, red and white, respectively. (**C**) Overall structure of MLE dsRBD_2_ in complex with R2H1. Left panel: cartoon view of the structure of MLE dsRBD_2_ in complex with R2H1. The critical residues belonging to regions 1, 2 and 3 required for dsRNA recognition are shown in stick mode and are colored in yellow, blue and green, respectively. Right panel: the electrostatic potential of the surface of the MLE dsRBD_2_–R2H1 complex, in which positively charged, negatively charged and neutral areas are represented in blue, red and white, respectively.

In the structure of the MLE dsRBD_1+2_–R2H1 complex, R2H1 forms a standard continuous A-form helix and the two dsRBDs bind on either side of the dsRNA (Figure 2A). Only individual dsRBDs, consisting of residues 1–80 of dsRBD_1_ (Figure 2B) and residues 165–247 of dsRBD_2_ (Figure 2C), were visible in the electron density map. The linker region connecting the two domains is not visible, which is consistent with the hypothesis that this linker is normally disordered (Figure 2A). R2H1 is a 55mer stem-loop-type helix RNA with a 13mer long ssRNA loop (Supplementary Figure S1B). Most nucleotides of the R2H1 are observed in the electron density map, except for part of the loop (Supplementary Figure S3A). In the crystal structure of the complex, part of the R2H1 loop structure (C135-U140) without protein protection was hydrolyzed, which is thought to stabilize the structure for better crystallization. The remaining bases of the loop (G141-C147) paired with the unhydrolyzed loop of the neighboring R2H1 in the next symmetry equivalent (Supplementary Figure S3A). The bases of the loop show elevated B factors, indicating the flexibility of this region. On the other side, R2H1 generated an RNA–RNA interface through end-to-end stacking with the next RNA (Supplementary Figure S3A). Therefore, the dsRNA forms a continuous helix extending through the crystal lattice (Supplementary Figure S3A).

Similar to all other members of the dsRBD family, MLE dsRBD_1_ and dsRBD_2_ share a conserved α1-β1-β2-β3-α2 core motif, in which the α1and α2 helices lie on a face of the three-stranded anti-parallel β sheets (Figure 2). The aliphatic side-chains and aromatic rings lying on α1, β1, β2 and α2 form a conserved hydrophobic core to maintain the stability of the whole domain. Each dsRBD spans one side of R2H1, constructing three crucial regions for dsRNA binding (Figure 2B and C). Regions 1 and 2 are inserted in two successive RNA minor grooves and region 3 contacts the intervening RNA major groove (Figure 2B and C). Region 1 of MLE dsRBD_2_ inserts below the apical loop of R2H1 (Figure 2C).

**Figure 3.**
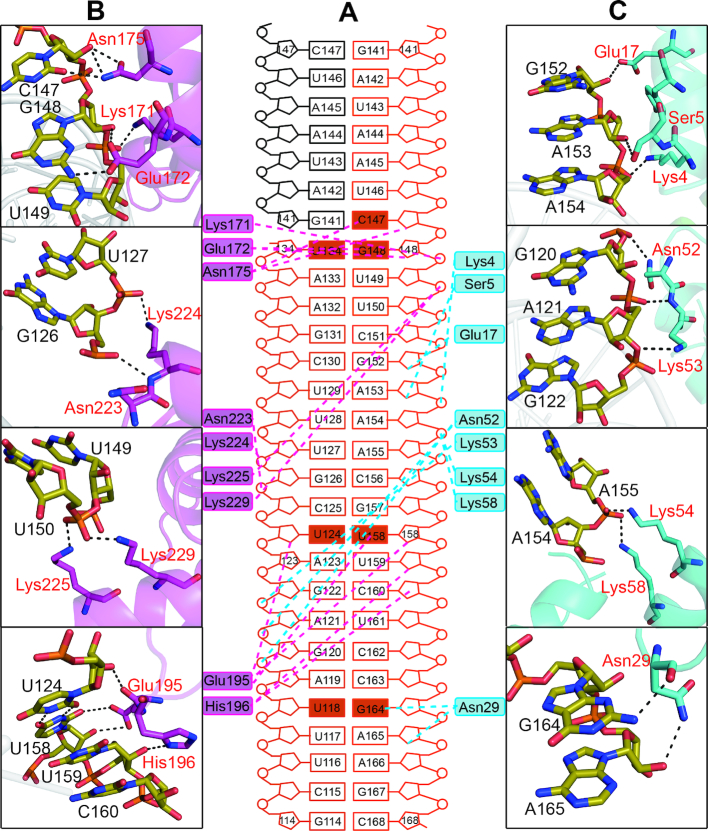
The interactions of MLE dsRBD_1+2_ with R2H1. (**A**) Schematic of R2H1 interactions with MLE dsRBDs, colored as described in Figure 2. Cyan and magenta dotted lines indicate contacts between MLE dsRBD_1_, dsRBD_2_ and R2H1, respectively. The wobble base pairs and mismatches involved in the recognition of dsRBDs by MLE are highlighted as solid rectangles. (**B**) Higher magnification views of individual interactions between MLE dsRBD_1_ (red) and R2H1 (black). Hydrogen bonds are indicated with black dotted lines. (**C**) Higher magnification views of individual interactions between MLE dsRBD_2_ (red) and R2H1 (black). Hydrogen bonds are indicated with black dotted lines.

In MLE dsRBD_1_, region 1 consists of the residues in the first α helix (Figure 2B). The side chains of Ser5 and Glu17 form hydrogen bonds with the 2′OH groups of the A153 and G152 ribose sugar rings, respectively (Figure 3A and C). Lys4 contacts phosphate groups in the minor groove via the positively charged side chain (Figure 3A and C). The loop joining β1 and β2 in region 2 interacts with the second minor groove (Figure 2B). This minor groove is widened to accommodate the wobble base-pairing of U118-G164. The peptide carbonyl of residue Asn29 binds to the amino group at position 2 of the G164 purine ring, which is specific to the guanine base. Additionally, the side chain of Asn29 forms a hydrogen bond with the next 2′OH group of the A165 ribose (Figure 3A and C). In region 3, the side chains of Asn52, Lys53, Lys54, and Lys58 interact with the phosphodiester backbone of both strands across the intervening major groove (Figures 2B, 3A and C).

MLE dsRBD_2_ shares a very similar structure with dsRBD_1_ (Supplementary Figure S2C). Region 1 (helix α1 of dsRBD_2_) inserts below the apical loop of R2H1. The U134-G148 wobble base pair and flexible loop enhance the accessibility of the nucleotides in this groove. The carbonyl group of Glu172 side chain and the amino group of Asn175 side chain form sequence-specific hydrogen bonds with the amino group at position 2 of the G148 purine ring and carbonyl group of the C147 base, respectively (Figure 3A and B). In addition, the side chains of Glu172 and Asn175 form hydrogen bonds with the ribose of G148 and C147, respectively, while Lys171 contacts the phosphodiester backbone of U149 (Figure 3A and B). The Glu195 and His196 residues in the loop connecting β1 and β2 are involved in region 2 (Figure 3A and B). This minor groove is widened to accommodate the base-pairing of the U124-U158 mismatch. The side chain of Glu195 forms a sequence-specific hydrogen bond with the carbonyl group of the U158 base. Moreover, the carbonyl group of the peptide backbone and side chain of Glu195 form direct hydrogen bonds with the 2′OH groups of U124 and U158 on the opposite strands, respectively. The imidazole cycle of His196 stacks on one ribose of C160 and forms hydrogen bonds with the 2′OH group of the previous ribose of U159 (Figure 3A and B). Similar to dsRBD_1_ region 3, Asn223, Lys224, Lys225 and Lys229 directly interact with the phosphodiester backbone of the major groove mainly via the positively charged side chain (Figures 2B, 3A and B).

### MLE dsRDB_1+2_–R2H1 recognition involves both shape and sequence specificity

R2H1 forms a standard A-form double helix characterized by a wide and shallow minor groove and a narrow and deep major groove (Figure 2A). This feature enhances the accessibility of the bases in minor groove, whereas the bases in major groove are more difficult to access. Therefore, we divided the interactions between MLE dsRBDs and R2H1 into two modes. First, an NKKxxxK motif in region 3 of both dsRBDs recognizes the major groove of R2H1. The side chains of the second Lys (Lys54 and Lys225) and the third Lys (Lys58 and Lys229) face one strand of R2H1 while the first Lys (Lys53 and Lys224) faces the other strand, crossing over the entire major groove (Figures 2B, C, 3B and C). This motif of MLE dsRBDs is the most conserved region in dsRBDs of diverse origins (Supplementary Figure S2A), ensuring a strict and specific recognition of the major groove width of the A-form RNA helix. Thus, the NKKxxxK motif in region 3 mediates shape recognition between MLE dsRBDs and R2H1.

**Figure 4.**
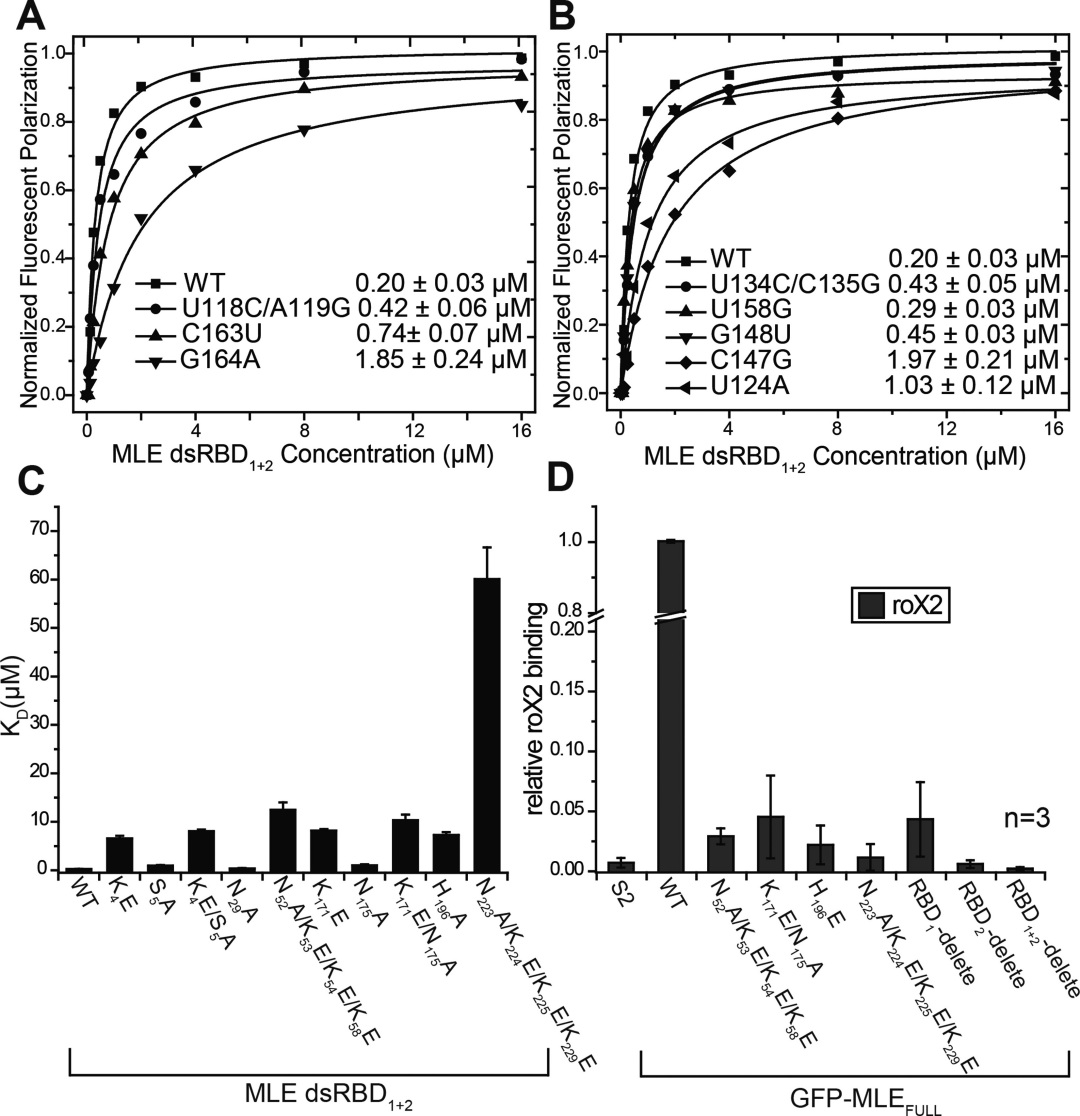
Effects of structure-based mutations. (**A** and **B**) The RNA-binding affinities of MLE dsRBD_1+2_ for R2H1-wild-type and indicated mutants, determined by FP experiments. *K*_d_ values and the corresponding standard errors were determined as described in the ‘Materials and Methods’ section. (**C**) The histogram of the *K*_d_ values shows the RNA-binding affinities of R2H1 for MLE RBD_1+2_-wild-type and indicated mutants, as determined by FP experiments. (**D**) RIP assays performed with GFP-tagged MLE_FL_-wild-type and indicated mutants. The abundance of roX2 RNA was quantified by qRT-PCR using specific primers for roX2. Relative roX2 enrichment (IP/input) in mutants was normalized to wild-type, while the relative roX2 enrichment (IP/input) in wild-type was set to 1.0. Data are presented as the means ± SD. *n* represents the number of experiments.

On the other hand, the residues in regions 1 and 2 of both MLE dsRBD_1_ and dsRBD_2_ form sequence-specific hydrogen bonds with the bases in the minor groove of R2H1 (Figure 3B and C). Interestingly, we identified specific contacts accompanying the wobble base pair or mismatch in R2H1. This recognition mode of MLE dsRBDs with R2H1 is similar to the base-specific interactions observed in ADAR2 bound to GluA2(R/G) (31). We performed FP assays by titrating MLE dsRBD_1+2_ against FAM-labeled R2H1 and its mutants to confirm the sequence-specific preference of MLE dsRBDs. Asn29 in region 2 of MLE dsRBD_1_ specifically recognized the U118-G164 wobble base pair of R2H1. The G164A mutation in R2H1 resulted in an ∼10-fold reduction in the binding affinity of MLE dsRBD_1+2_ for the R2H1 mutant compared with that observed for wild-type R2H1 (Figure 4A and Supplementary Table S1). Meanwhile, following the replacement of U118-G164 of R2H1 by a Watson–Crick C118-G164 pair, the binding affinity of MLE dsRBD_1+2_ for R2H1 mutant was reduced by merely 2-fold (Figure 4A and Supplementary Table S1). These results confirmed that Asn29 in region 2 of MLE dsRBD_1_ recognizes the sequence rather than the shape of the U118-G164 wobble base pair. Similarly, the residues in region 1 of MLE dsRBD_2_ sequence-specifically recognize C147 in the loop of R2H1. The C147 mutation in R2H1 resulted in a ∼10-fold reduction in the binding affinity of MLE dsRBD_1+2_ for R2H1 mutant. However, following the replacement of U134-G148 wobble base pair and C135-C147 mismatch by CG pairs, the binding affinities of MLE dsRBD_1+2_ for the R2H1 mutant were reduced by merely 2-fold (Figure 4B and Supplementary Table S1). However, the recognition of the U124-U158 mismatch by Glu195 in region 2 of MLE dsRBD_2_ is different from the recognition of U118-G164 by Asn29. In the presence of the U158G mutation, the binding affinity of MLE dsRBD_1+2_ for the R2H1 mutant is almost identical to that observed for the wild-type R2H1. However, the U124A mutation caused an ∼5-fold reduction in the binding affinity (Figure 4B and Supplementary Table S1). The U124A mutation may induce the movement of the base of U158 and affect the interaction between U158 and Glu195.

Based on these results, both shape-specific interactions and sequence-specific interactions are important for the binding affinity of MLE dsRBD_1+2_ for R2H1 and the specific recognition of R2H1 is mediated by both MLE dsRBD_1_ and MLE dsRBD_2_.

### Structure-based mutations affect the ability of MLE to bind to roX2 *in vitro*

To determine whether these key residues of MLE dsRBDs were required for the binding of R2H1 *in vitro*, we introduced alanine and glutamate mutations into MLE dsRBDs. Subsequently, we performed FP assays to measure the binding affinities of different MLE dsRBDs mutants for R2H1. As expected, the introduction of quadruple mutations into MLE dsRBD_1+2_, which destroy the major groove recognition site (NKKxxxK motif), almost abolished R2H1 duplex binding. The effect was more pronounced with the N223A/K224E/K225E/K229E mutation (Figure 4C and Supplementary Table S1). Similarly, the reverse-charge substitutions of positively charged residues involved in the recognition of the R2H1 minor groove (K4E, K171E or H196E single mutations, K4E/S5A or K171E/N175A double mutations), also significantly reduced R2H1 duplex binding (Figure 4C and Supplementary Table S1). Although, Asn29 binds to the G164 purine ring through its peptide carbonyl group, the introduction of an N29A single mutation into MLE dsRBD_1+2_ showed comparable R2H1 duplex-binding affinity to wild-type MLE dsRBD_1+2_ (Figure 4C and Supplementary Table S1). In contrast, the E172A and E195A single mutations resulted in a slightly enhanced interaction with R2H1 (Supplementary Table S1). Thus, E172 and E195 may interact with R2H1, not only to specifically recognize the sequence in minor groove of R2H1 but also to make-up electrostatic repulsion with the negatively charged phosphate backbone. CD spectra analyses confirmed that all mutants of MLE dsRBDs maintain a similar secondary structure composition that of wild-type MLE dsRBDs (Supplementary Figure S1C).

### Mutations in dsRBDs significantly reduce the endogenous roX2 affinity of MLE

The *in vitro* assays revealed that both dsRBDs are important for dsRNA interaction, and the mutations of key residues in MLE dsRBDs drastically affect the interaction with R2H1. Plasmids expressing the full-length, wild-type MLE with GFP fused at N terminus (GFP-MLE_FL_), as well as the mutants, were transfected into *Drosophila* S2 cells to assess the effects of MLE dsRBDs deletion or mutations of key residues in MLE dsRBDs on the MLE function *in vivo*. Native RNA-immunoprecipitation (RIP) assays were performed to measure the interactions between GFP-MLE_FL_ and roX2 *in vivo*. RIP assays were performed using the GFP-Trap beads and the amount of endogenous roX2 bound by GFP-MLE_FL_ was detected using qRT-PCR. Based on RIP results, wild-type GFP-MLE_FL_ efficiently retrieved the endogenous roX2 RNA (Figure 4D), but not the Pka RNA from an autosomal gene (Supplementary Figure S4A), although in the S2 cell, the levels of Input roX2 and Pka RNA were similar (Figure 4D and Supplementary Figure S4A). Compared with the wild-type GFP-MLE_FL_, the introduction of single or multiple point mutations severely compromised roX2 binding *in vivo*, particularly the mutations in region 3 of MLE dsRBD_2_ (Figure 4D and Supplementary Figure S4A). Moreover, the deletion of either MLE dsRBD almost completely abolished the interaction between MLE and roX2 *in vivo*, particularly the deletion of MLE dsRBD_2_ or dsRBD_1+2_ (Figure 4D and Supplementary Figure S4A). Furthermore, MLE dsRBD_2_ played a more important role in the MLE_FL_–roX2 interaction than MLE dsRBD_1_. In summary, based on the RIP data, structure-based mutations or deletion of MLE dsRBDs significantly affected the ability of MLE to bind roX2 *in vivo*, corroborating the *in vitro* interaction data.

### A decrease in the affinity of MLE for roX results in male lethality and disrupts dosage compensation to different extents

We generated three *mle* mutant alleles of *Drosophila melanogaster* using precise gene editing based on homologous recombination induced by CRISPR/Cas9 system to determine whether decreasing the affinity of MLE for roX2 affected the viability of male flies and dosage compensation *in vivo* (37). We acquired two *mle* alleles encoding mutant MLEs with four key amino-acid residues substituted in dsRBD_1_ (*mle^subRBD1^*, N52A/K53E/K54E/K58E) and dsRBD_2_ (*mle^subRBD2^*, N223A/K224E/K225E/K229E), as well as a *mle^ΔRBDs^* allele with a precise deletion of the entire region encoding the two dsRBDs (Supplementary Figure S5A). None of female flies with these alleles showed any mutant phenotype or decreased viability compared with that observed in *wild-type* or heterozygous females.

**Figure 5.**
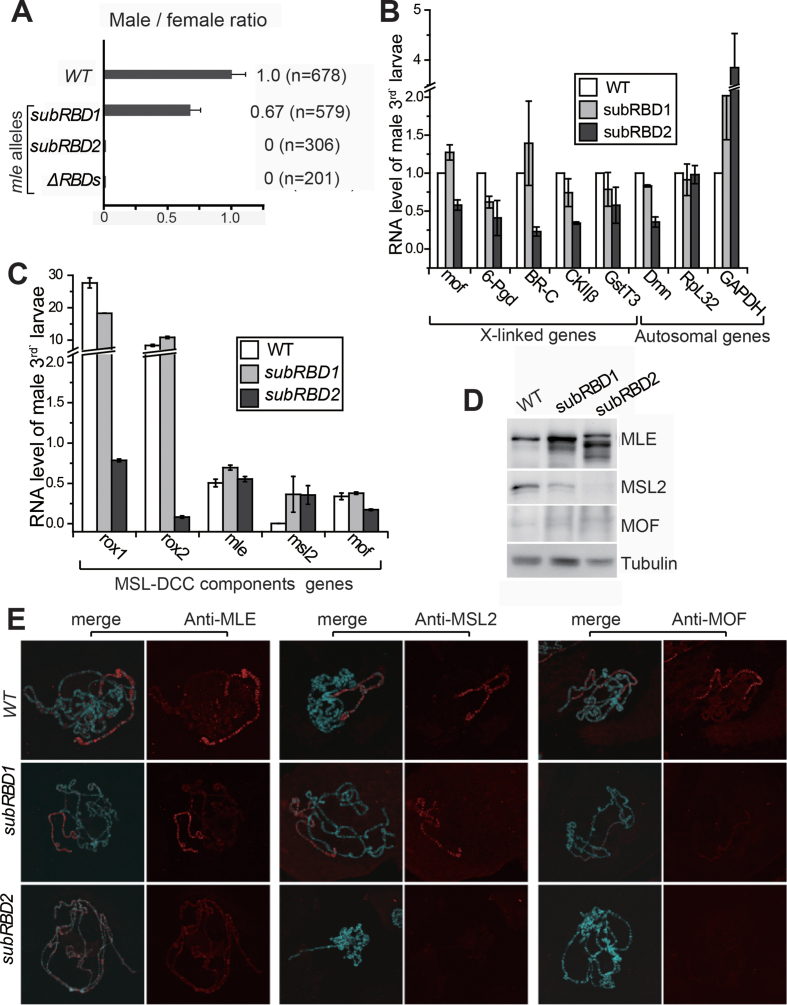
Structure-based mutations in *mle* dsRBDs result in male lethality and disrupt dosage compensation to different extents. (**A**) A weaker RNA affinity of MLE was linked to decreased viability in male flies. The ratio of males/females for each mutant compared with that of *wild-type* was used to describe male viability. (**B**) qPCR analysis of genes on the X chromosome and on autosomes in male *wild-type, mle^subRBD1^* and *mle^subRBD2^* third instar larvae. Error bars are defined as the standard deviation (s.d.) of triplicate experiments. The expression level of each gene was normalized to *wild-type* and set to 100% for each gene in *wild-type*. (**C**) qPCR analysis of MSL-DCC component genes in third instar of *wild-type, mle^subRBD1^* and *mle^subRBD2^* larvae. Error bars are defined as the s.d. of triplicate experiments. The expression level of each gene was normalized to the autosomal gene *Pka*. (**D**) The levels of the MLE, MSL2 and MOF proteins in male *wild-type, mle^subRBD1^* and *mle^subRBD2^* third instar larvae. (**E**) Localization of the MSL-DCC components on the polytene chromosomal spreads of male third instar larvae carrying the *wild-type* and *mle* mutant alleles. Confocal images of immunostaining for MLE, MSL2 and MOF visualized using an Alexa Fluor 594-conjugated secondary antibody (red) and DNA counterstaining with DAPI are shown.

First, we tested the male/female ratio of each genotype to assess male viability. No adult male homozygous for *mle^subRBD2^* or *mle^ΔRBDs^* survived, while the male/female ratio of *mle^subRBD1^* adults decreased to 0.67 compared with *wild-type* adults (Figure 5A). Moreover, we further investigated these flies at earlier stages. Most males with the *mle^subRBD2^* genotype died during pupation, since the male/female ratio was 0.9 in wandering larval stage (*n* = 237), with numerous dead pupae observed on the vial wall. Due to the lack of dsRBDs, *mle^ΔRBDs^* males survived only to the early third instar larval stage. Thus, any decrease in the RNA affinity of MLE induced by either point mutations or the deletion of dsRBDs impaired the function of MLE and caused male lethality at different stages.

Dosage compensation involves the doubling of the transcription of X-linked genes in male *Drosophila*. Therefore, we detected the RNA levels of various X-linked genes using qRT-PCR to measure the impacts of these MLE mutations on dosage compensation. We analyzed expression levels of the X-linked dosage-compensated genes *6-pgd, BR-C, CKIIβ* and *GstT3* in males, and the autosomal genes *Pka, Dmn, RpL32* and *GAPDH* served as controls.

In male third instar larvae, the *mle^subRBD2^* mutations exerted a stronger effect than the *mle^subRBD1^* mutations. Compared with the RNA levels in *wild-type* larvae, in *mle^subRBD1^* larvae, the expression of X-linked genes was only slightly increased or reduced. In contrast, the expression of these genes was reduced by half in *mle^subRBD2^* larvae (Figure 5B). In *mle^subRBD1^* larvae, the expression of autosomal genes was not substantially affected, but in *mle^subRBD2^* larvae, the expression of *Pka* and *RpL32* remained unaffected, whereas that of *Dmn* and *GAPDH* was reduced and increased respectively. We also collected a number of single second instar larvae (72 h after egg-laying) and used the balancer *CyO-GFP* and PCR of the *ary* gene on the Y chromosome to precisely select the male larvae homozygous for *mle^ΔRBDs^* for RNA extraction. In second instar male *mle^ΔRBDs^* larvae, the RNA levels of all X-linked genes were reduced by ∼50% compared to wild-type levels (Supplementary Figure S5B), which is not surprising considering the total loss of dsRBDs in MLE. Based on these results, a disruption in the RNA binding ability of MLE via the deletion of the tandem dsRBDs or the substitution of key residues impeded dosage compensation in male flies.

Additionally, the RNA levels of MSL-DCC component genes displayed different patterns of change in flies carrying these *mle* alleles (Figure 5C and Supplementary Figure S5C). First, the levels of *roX1* and *roX2* were decreased in *mle^subRBD2^* and *mle^ΔRBDs^* larvae to <10% of the level in *wild-type* larvae, but no change was detected in *mle^subRBD1^* larvae. The levels of the *mle* RNA remained similar to *wild-type* in the larvae carrying the two *mle^subRBD^* alleles, but were substantially increased in *mle^ΔRBDs^* larvae. The *msl2* RNA levels were drastically increased in larvae carrying all three alleles. The levels of *mof* were slightly decreased in *mle^subRBD2^* and *mle^ΔRBDs^* larvae.

We further determined the levels of the MLE, MSL2 and MOF proteins in third instar larvae samples through western-blotting to investigate potential changes in the expression of these MSL-DCC proteins (Figure 5D). MLE level was slightly elevated in *mle^subRBD1^* males. However, in *mle^subRBD2^* males, MLE level was substantially elevated and showed changes in band migration, indicating different protein modifications versus the wild-type protein. To our surprise, a lower MSL2 level was detected in *mle^subRBD1^* males and an almost undetectable signal was observed in *mle^subRBD2^* males on western -blots, although the *msl2* RNA level was increased in males carrying these two alleles. Notably, only weak bands were observed for MOF (Figure 5D).

Based on these results, the components of MSL-DCC exhibited extensive changes as a consequence of the decreased binding affinity of MLE for dsRNA. The *mle^subRBD1^* allele only induced mild changes. However, in *mle^subRBD2^* males, the RNA level of *roX* was substantially decreased, while the level of the MLE protein was increased and its modification was altered. The most interesting finding was that the level of the MSL2 protein was decreased, despite the RNA level is increased compared with that of *wild-type* flies. The *mle^subRBD2^* mutation resulted in extensive changes similar to *mle^ΔRBDs^* .

Subsequently, we visualized the MSL-DCC using immunostaining with antibodies against MLE, MSL2 and MOF in the salivary gland of the male third instar larvae to investigate whether the MSL-DCC incorporating these mutant MLE proteins still exhibited X-chromosome-specific localization (Figure 5E). Compared with the *wild-type* samples (Figure 5E, top panel), *mle^subRBD1^* displayed relatively normal signals for MLE and MSL2, and weaker staining for MOF (Figure 5E, middle panel). In *mle^subRBD2^* samples, MLE proteins were present as numerous bands and displayed weak staining on all chromosomes, but completely lost the X-chromosome-specific localization observed in *mle^subRBD1^* and *wild-type* flies. Moreover, low signals were observed for MSL2 or MOF on chromosomes (Figure 5E, bottom panel). Thus, a decrease in the affinity of MLE proteins for dsRNA largely disrupted the accumulation of the MSL-DCC on X-chromosome, in which dsRBD_2_ plays major roles compared with dsRBD_1_, consistent with their RNA affinity.

## DISCUSSION

Previous studies have shown that dsRBDs are well-characterized domains that bind dsRNA modules (26,39). In *Drosophila* dosage compensation, the N-terminal dsRBDs of MLE play an essential role in recognizing the natural dsRNA target. Thus, MLE performs its ATPase and helicase functions to unwind R2H5 (roX2 helix5) for incorporation into a productive MSL-DCC (12,19,23). In our study, we determined the structure of MLE tandem dsRBDs with a natural dsRNA target at 2.90 Å and revealed that the specific recognition of roX2 dsRNA by MLE dsRBDs is indispensable for the assembly of the MSL-DCC in *Drosophila* dosage compensation.

In the structure of the MLE_core_–U_10_–ADP–AlF_4_ complex, the auxiliary domains of MLE_core_ coordinate with RecA domains to recognize the UxUUU motif (20). The structure of the MLE_core_–U_10_–ADP–AlF_4_ complex provides insights into the mechanisms of ATP-dependent MLE helicase activity and the ssRNA binding status (20). As previously reported, the vast majority of RNA helicase core domains do not display sequence or structural specificity (40). Meanwhile, our reported structure of the MLE dsRBD_1+2_–R2H1 complex provides complementary evidence and a deeper understanding of the mechanism by which MLE recognizes the roX RNA through the two additional N-terminal dsRBDs in the initial step of roX RNA remodeling.

**Figure 6.**
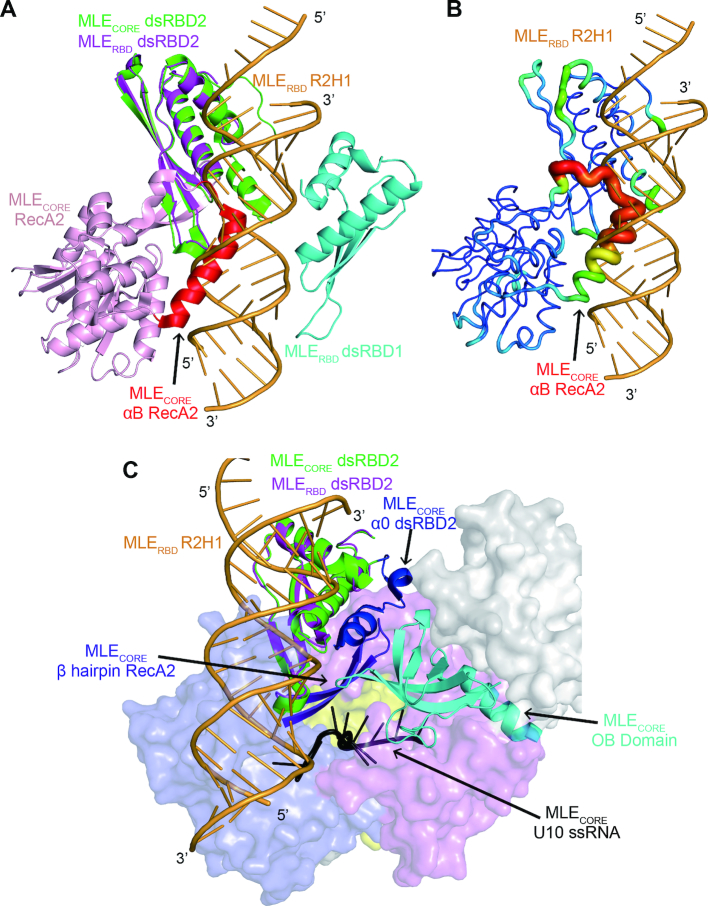
Superposition of the MLE dsRBD_1+2_–R2H1 complex and MLE_core_–U_10_–ADP–AlF_4_ complex. (**A**) The superposition of the MLE dsRBD_1+2_–R2H1 complex with the structure of the MLE_core_–U_10_–ADP–AlF_4_ complex shows that helix α_B_ of RecA_2_ (highlighted in red) in MLE_core_ possesses a physical barrier with the dsRNA–R2H1 in our complex. The MLE dsRBD_1+2_–R2H1 complex is colored as described in Figure 2A. For clarity, the MLE_core_–U_10_–ADP–AlF_4_ complex only shows the structure of dsRBD_2_ (green) and the RecA_2_ domain (light pink). (**B**) The helix α_B_ of RecA_2_ in MLE_core_ shows elevated B factors compared with those of other regions in MLE_core_. For clarity, the MLE dsRBD_1+2_–R2H1 complex only shows R2H1, whereas the MLE_core_–U_10_–ADP–AlF_4_ complex only shows the dsRBD_2_ and the RecA_2_ domain in B-factor mode in PyMOL. (**C**) N-terminal helix (α_0_) of MLE_core_ dsRBD_2_ is involved in the formation of the ssRNA-binding channel for poly-U. The MLE_core_ ssRNA-binding channel formed by the α_0_ of dsRBD_2_ (blue), the OB domain (cyan) and the hairpin of RecA_2_ (purple). The U_10_RNA is colored in black. For clarity, the MLE dsRBD_1+2_–R2H1 complex only shows the dsRBD_2_ and R2H1. The MLE_core_ ssRNA-binding channel and U_10_RNA are shown as cartoons, whereas the other domains of MLE_core_–U_10_RNA complex are shown as surfaces with different colors.

The superposition of our MLE dsRBD_1+2_–R2H1 structure with MLE_core_–U_10_–ADP–AlF_4_ structure, which both contain dsRBD_2_, shows that helix α_B_ of RecA_2_ in MLE_core_ poses a physical barrier with the dsRNA in our complex (Figure 6A and B). The helix α_B_ of RecA_2_ is a distinctive helical insertion of RecA_2_ located close to the entry of the ssRNA-binding channel (20). However, the helix α_B_ of RecA_2_ shows obviously elevated B-factors compared with those of other regions of MLE_core_, indicating that the helix α_B_ of RecA_2_ is flexible when MLE only binds an ssRNA (Figure 6A and B). The helix α_B_ of RecA_2_ appears to undergo a conformational change as a switch when MLE binds to a dsRNA or an ssRNA. The superposition presents a possible model of MLE binding to both dsRNA and ssRNA. In the MLE_core_–U_10_–ADP–AlF_4_ complex, the helix α_B_ of RecA_2_ has few contacts with other parts of MLE_core_ and exhibits like a ‘closed’-like state, protecting the entry of the ssRNA-binding channel. When the MLE dsRBDs recognize and bind to the dsRNA target, the helix α_B_ of RecA_2_ undergoes a conformational switch to an ‘open’ state to accommodate the dsRNA. Subsequently, MLE_core_ exerts its ATPase and helicase activity to unwind the roX RNA and facilitate the assembly of the MSL-DCC.

Furthermore, the structure of the MLE_core_–U_10_–ADP–AlF_4_ complex shows that MLE dsRBD_2_ contains an unusual structural element, an N-terminal helix (α_0_), which was not observed in our crystal structure (20). The α_0_ helix of MLE dsRBD_2_ does not appear to be involved in the recognition of dsRNA. The superposition of our MLE dsRBD_1+2_–R2H1 complex with the structure of MLE_core_–U_10_RNA complex (Figure 6C) shows that the N-terminal helix (α_0_) of dsRBD_2_ faces the helicase core domain and has little contact with the dsRNA, confirming that the α_0_ of MLE dsRBD_2_ is not involved in the recognition of dsRNA. As shown in the study by Elena Conti, the α_0_ helix of dsRBD_2_ interacts with the OB domain and the hairpin structure of RecA_2_, stabilizing the MLE helicase core and forming the 5′ portion of the ssRNA-binding channel (20). Thus, the additional α_0_ helix in dsRBD_2_ is essential for the binding of ssRNA, but not dsRNA.

MLE is a well-characterized ATP-dependent DEXH box dsRNA helicase. The N-terminal tandem dsRBDs of MLE play essential roles in specifically locating the roX RNA target. As shown in our previous study, MLE dsRBD_1_ and MLE dsRBD_2_ share a similar structure, with a root-mean-square (r.m.s.) deviation for Cα atoms of 1.038 Å. Moreover, their binding mode with R2H1 is analogous (Supplementary Figure S2C). Although MLE dsRBD_1_ and MLE dsRBD_2_ share only 20% sequence identity (Supplementary Figure S2B), the two dsRBDs show only slight differences in structures (Supplementary Figure S2C). However, the binding affinity of MLE dsRBD_2_ for R2H1 is ∼10-fold higher than that of MLE dsRBD_1_ (Figure 1D). Notably, our structural analysis also showed significant contributions of the basic residues from regions 1 and 2 to the differences in binding affinity. Lys171, Glu172, Asn175 in α1 and Glu195, His196 in region 2 of dsRBD_2_ have longer side chains and form a cluster to ensure tighter insertion into the minor groove of R2H1 than Lys4, Ser5 and Asn29 in dsRBD_1_, which face the minor groove of R2H1 in a spread and non-compact manner (Figures 2 and 3). Furthermore, MLE dsRBD_2_ binds to the major groove in region 3 more tightly than dsRBD_1_. The *K_d_* values revealed a ∼5-fold lower RNA-binding affinity of the MLE RBD_1+2_-N223A/K224E/K225E/K229E mutant for R2H1 than the MLE RBD_1+2_-N52A/K53E/K54E/K58E mutant (Figure 4C and Supplementary Table S1). These structural differences between MLE dsRBD_1_ and MLE dsRBD_2_ might explain the different binding affinities for R2H1.

MLE dsRBD_1_ and dsRBD_2_ exhibit substantial differences in binding affinity for roX2 RNA, which are quite different from the homologous dsRBDs structures of the human RNA helicase A-DHX9. DHX9 is a RISC-loading factor that also contains two tandem dsRBDs at its N-terminus enabling it to interact with various small dsRNA and participate in the RNA silencing pathway (30). Moreover, DHX9 dsRBDs interact with the essential components of complexes involved in different cellular processes (41–43). Various functions of DHX9 result in the high affinity but less specificity for dsRNA. Both dsRBDs of DHX9 have the capacity to interact with the siRNA duplex (30). However, MLE dsRBD_1_ exhibits a weak interaction with the substrate alone *in vitro*, which seems to be inconsistent with DHX9 dsRBD_1_. Based on the sequence alignment, dsRBD_1_ is more conserved than dsRBD_2_ (Supplementary Figure S2A). The alignment of MLE dsRBDs with the structure of DHX9 dsRBDs shows that the r.m.s. deviation for Cα atoms of the two dsRBDs is only 0.515 Å and 0.706 Å, respectively (Supplementary Figure S3B). Moreover, the residues of the two dsRBDs involved in recognizing dsRNA are similar (Supplementary Figure S3B). However the structural analysis showed the failure of residues in region 1 of MLE dsRBD_1_ to insert into and specifically recognize the minor groove of dsRNA. Moreover, we compared our complex structure with other dsRBD-dsRNA complexes (Supplementary Figure S3C–E) (44–46). Based on the comparison, the orientations of helix α1 with respect to helix α2 in these dsRBDs exhibit considerable differences. As known, different orientations of helix α1 result in different ‘register length’ of different dsRBDs, which in turn influence the binding ability and special recognition of dsRBDs for dsRNA targets (26,44). In addition, certain dsRBDs adopt remarkable extension helices (α3 of Rnt1p dsRBD and α0 of TRBP dsRBD_1_) to stabilize the helix α1 (Supplementary Figure S3D and 3E) (45,46). Collectively, the orientations and residues involved in dsRNA recognition of helix α1 play an indispensable role in ensuring the effective binding of dsRBDs for dsRNA. In our study, MLE dsRBD_1_ was shown to play an indispensable role in the recognition of R2H1 and contributes to a higher binding affinity owing to its synergistic effect with MLE dsRBD_2_. This finding indicated that dsRBD_1_ may also be essential for the sequence-specific interaction with dsRNA. According to our RIP assays, deletion of dsRBD_1_ results in a severe compromise in roX2 binding *in vivo*. Moreover, MLE lacking dsRBD_1_ failed to target the X-chromosome territory in male SF4 cells (32). The substitutions of the four key residues resulted in partial male lethality and failure of MOF localization on the X chromosome of male larvae (Figure 5E). MLE dsRBD_1_ may not only participate in dsRNA recognition but also interact with other structures within the MSL-DCC. The RHA dsRBD_1_ and the proline-rich domain of RHA form a composite channel to recognize a specific dsDNA (47). Thus, the MLE dsRBD_1_ may interact with other components of the MSL-DCC, such as MSL2 which contains a proline-rich domain at its C-terminus. In addition, MLE plays a role in RNA splicing, similar to ADAR site-selective A-to-I editing (48). The recognition mode of MLE dsRBDs for R2H1 is similar to base-specific interactions observed in ADAR2 bound to GluA2(R/G). The specific recognition of MLE dsRBDs for dsRNA ensures that MLE accurately executes its functions.

Additionally, in this study the structure-based mutations produced *ml*e alleles with a partial loss of function. These mutant MLEs with lower RNA affinity impaired dosage compensation in male flies to various extents. Moreover, they indicated an association between the affinity of dsRBDs for roX2 and the severity of the mutant phenotypes. This was displayed through the localization of DCC components, the expression levels of X-linked genes and the mortality of male flies. These are well-known consequences of impaired dosage compensation (15,49,50). Loss of the RNA binding affinity of MLE, as observed in *mle^subRBD2^* and *mle^ΔRBDs^* males, results in drastic reductions in the levels of roX RNAs (>10-fold). Evidence previously reported that MLE may directly bind to the upstream genomic region of the roX2 gene and promote transcription of roX2 (51). Moreover, several components of DCC (including MSL1, 2, 3 and MLE) are required for the enhancement of roX RNA transcription, which is mediated by the MSL-binding sites (DHS, DNase I hypersensitive site) (52). The decreased RNA levels of roX observed in *mle^subRBD2^* and *mle^ΔRBDs^* indicate that the binding ability of MLE for roX RNA is also essential for its role in inducing the transcription of roX RNA.

The mutant MLE protein was not bound to the X chromosome in *mle^subRBD2^* males and failed to form complex with other components of DCC. Immunoblotting analysis revealed that these mutant proteins were displayed as multiple bands of different migration from those in wild-type samples, which could be partially caused by degradation of the dysfunctional proteins, and we also proposed that the post-translational modifications may be involved in the function of MLE in dosage compensation (Figure 5D).

As the only male-specific component, the expression of MSL2 protein is strictly suppressed by SXL and its cofactors to maintain dosage compensation ‘inactive’ in female flies (53,54). In male flies, the abundance of MSL2 protein may also be regulated. In the present study, we found that in *mle^subRBD^* male larvae, the levels of the MSL2 protein were largely reduced, despite an increase in the levels of *msl2* RNA. The normal expression of MSL2 was dependent on MLE in a direct or indirect manner. It has been reported that the non-chromatin-associated free MSL complex binds to and retains *msl2* RNA in the nucleoplasm, which may reduce the number of *msl2* transcripts available for export and translation through a negative feedback mechanism (50). As the only male-specific component, the abundance of MSL2 protein is strictly regulated to achieve the proper extent of dosage compensation.

## DATA AVAILABILITY

Atomic coordinates and structure factors for the reported crystal structure have been deposited with the Protein Data Bank under accession number 5ZTM.

## Supplementary Material

Supplementary Data
