# Supplementary material for: Structural insights reveal the specific recognition of roX RNA by the dsRNA-binding domains of the RNA helicase MLE and its indispensable role in dosage compensation in Drosophila
Source: Nucleic Acids Res. 2019 Jan 15;47(6):3142–57. doi: 10.1093/nar/gky1308 (PMC6451107; doi:10.1093/nar/gky1308)
Supplement: Supplementary Data [file gky1308_supplemental_files.pdf]

Supplementary Materials

Supplementary Figure S1

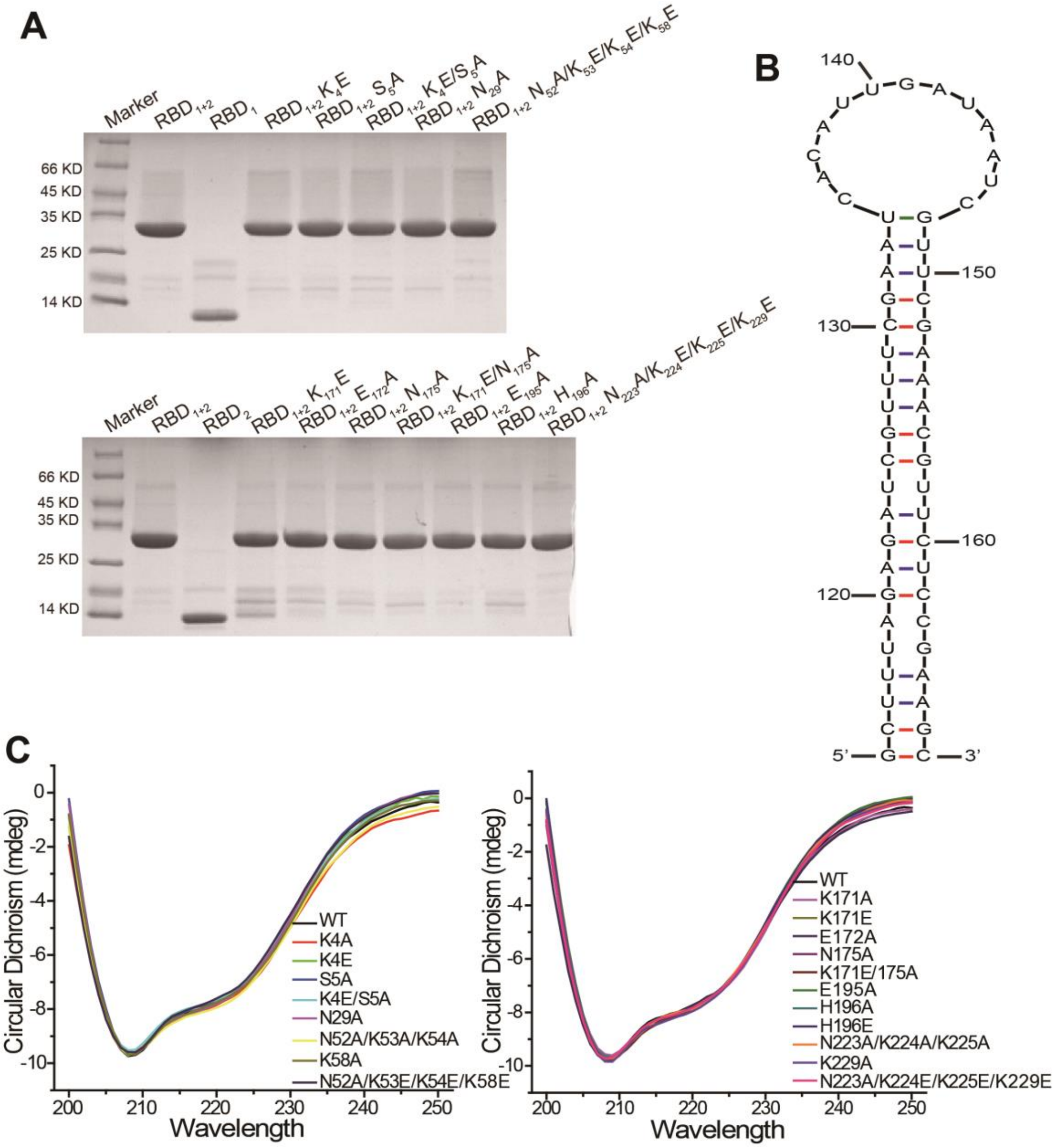

Supplementary Figure S2.

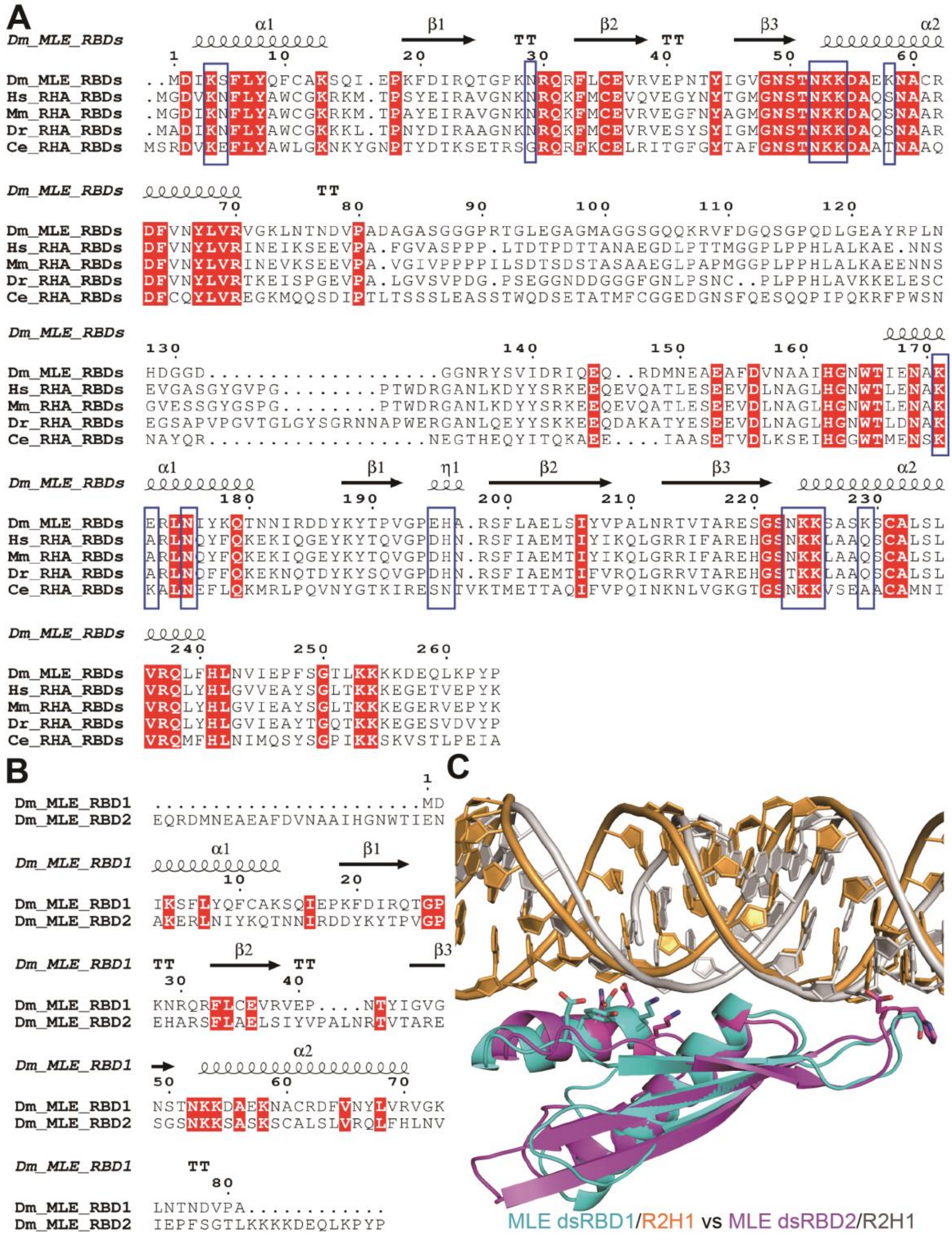

Supplementary Figure S3.

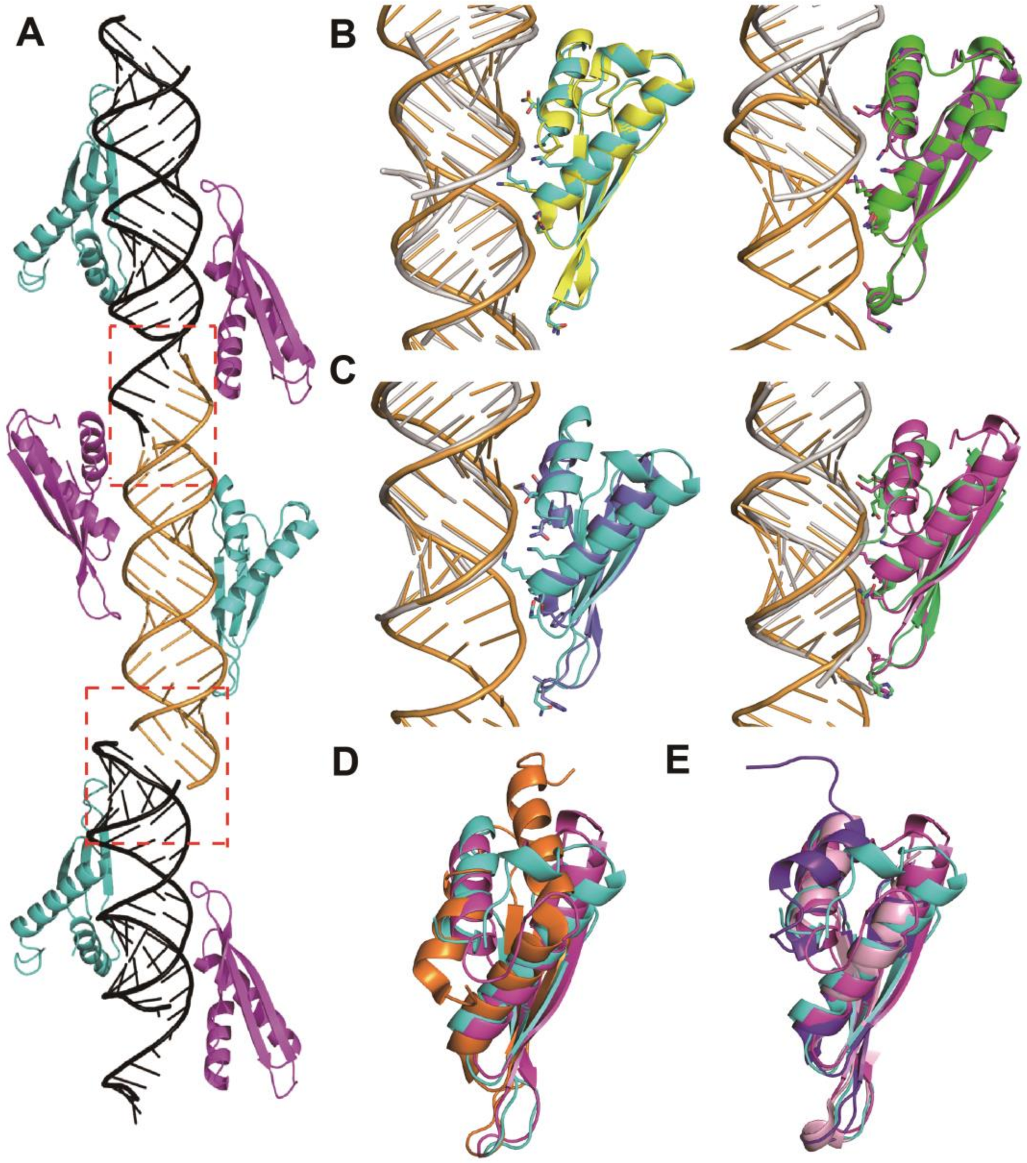

Supplementary Figure S4.

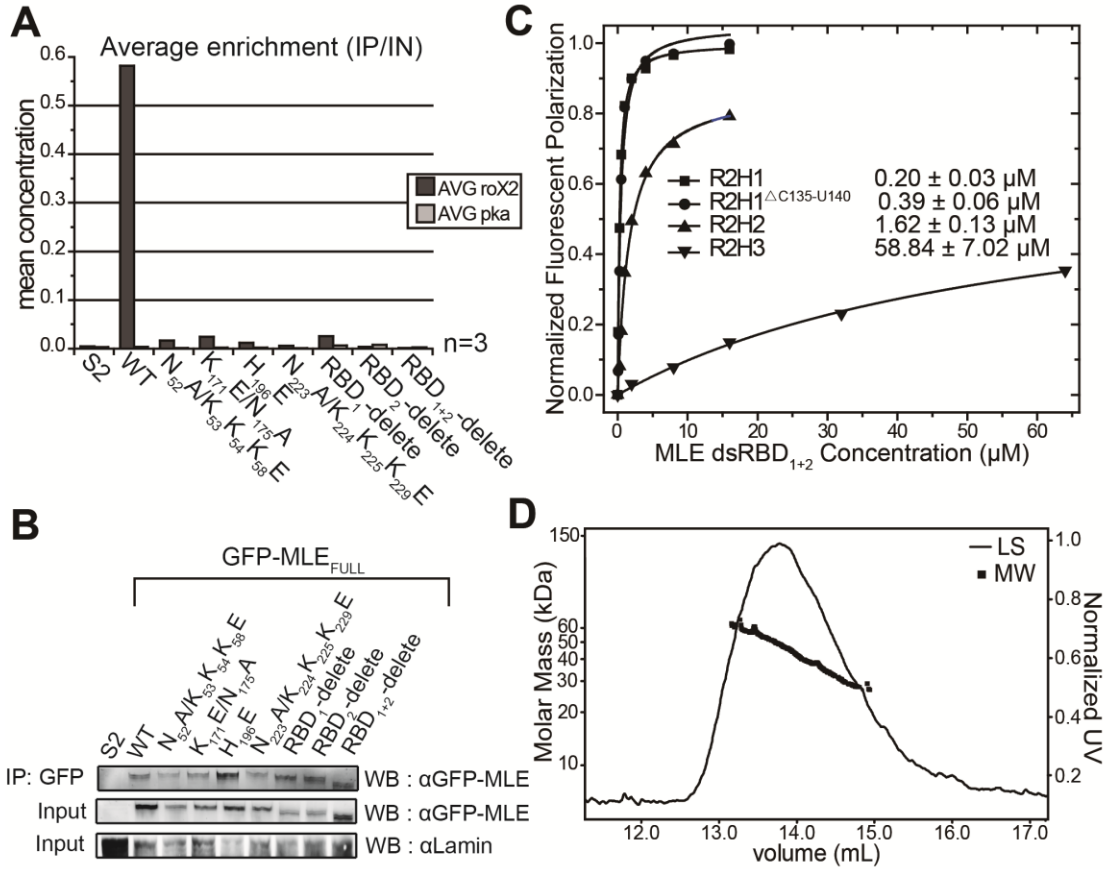

Supplementary Figure S5.

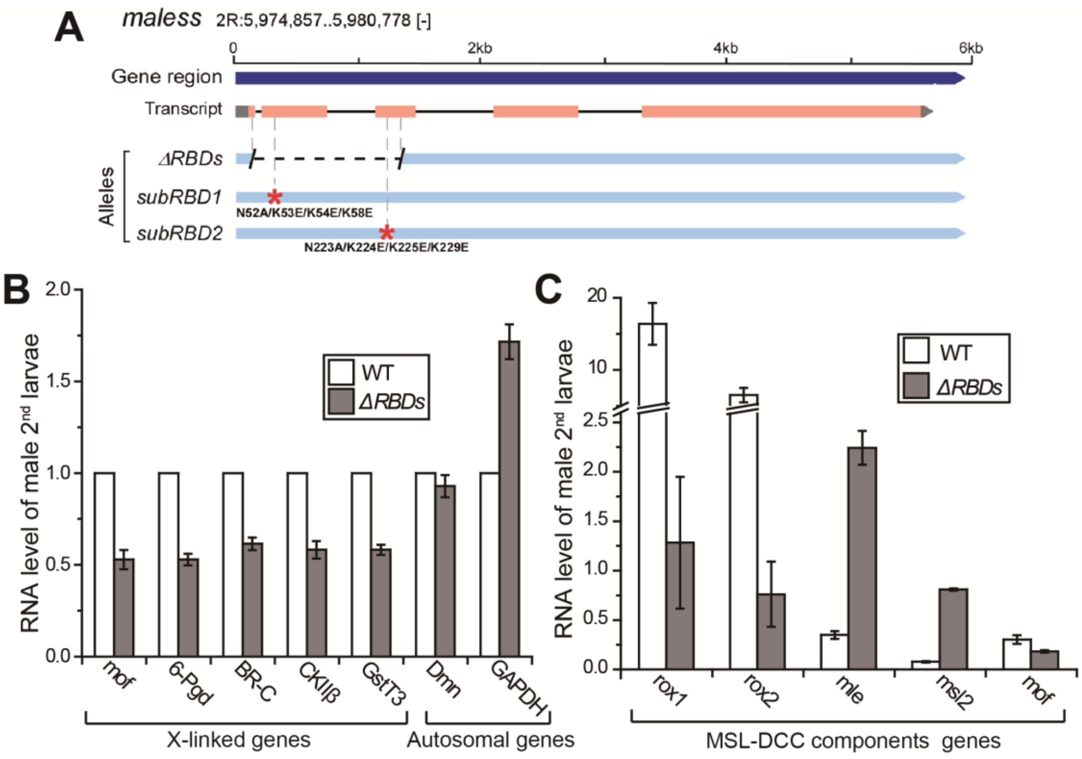

Supplemental Figure Legends

Supplementary Figure S1. (A) SDS-PAGE gel of different MLE dsRBDs constructs and MLE dsRBD$_{1+2}$ mutants at the same concentrations. (B) Overview of the first stem-loop of the roX2-R2H1 construct used in RNA binding assays and co-crystallization with MLE dsRBDs. (C) CD spectra of MLE dsRBD$_{1+2}$ and its mutants.

Supplementary Figure S2. Evolutionary conservation of MLE dsRBD$_{1+2}$. (A) Sequence alignment of MLE dsRBD$_{1+2}$ with various dsRBD proteins from different organisms: *Drosophila melanogaster* (*Dm*) MLE and the orthologous RNA helicase A (RHA) from *Homo sapiens* (*Hs*), *Danio rerio* (*Dr*) and *Caenorhabditis elegans* (*Ce*). The secondary structure elements of MLE dsRBDs are shown above the sequence, as helices (for α-helices) and arrows (for β-strands). The critical residues required for the recognition of the dsRNA are highlighted with dashed blue boxes. (B) Sequence alignment of MLE dsRBD$_1$ and dsRBD$_2$ present in the crystal structure. (C) The superposition of the MLE-dsRBD$_1$-R2H1 complex (MLE dsRBD$_1$ colored in cyan and R2H1 colored in bright orange) and MLE-dsRBD$_2$-R2H1 complex (MLE dsRBD$_2$ colored in magenta and R2H1 colored in gray). The critical residues required for the recognition of the dsRNA minor groove are shown in stick mode, respectively.

Supplementary Figure S3. (A) Symmetry and stereo views of the MLE dsRBD$_{1+2}$-R2H1 complex in the crystal structure. The bases of the loop (G$_{141}$-C$_{147}$) paired with the unhydrolyzed loop of the neighboring R2H1 in the next symmetry equivalent are highlighted with a dashed red box (upper panel). On the other side R2H1 generated an RNA-RNA interface by stacking end-to-end with the next RNA, as highlighted with a dashed red box (lower panel). (B and C) Left panel: Superposition of the MLE dsRBD$_1$-R2H1 complex (MLE dsRBD$_1$ and R2H1 colored in cyan and bright orange, respectively) with the hsRHA-dsRBD$_1$-GC10 duplex complex (hsRHA dsRBD$_1$ and GC10 colored in yellow and gray, respectively) and NF90-dsRBD$_1$–18mer dsRNA complex (NF90 dsRBD$_1$ and 18mer dsRNA colored in slate and gray, respectively). Right panel: Superposition of the MLE-dsRBD$_2$-R2H1 complex (MLE dsRBD$_2$ and R2H1 colored in magenta and bright orange, respectively) with the hsRHA-dsRBD$_2$-GC10 duplex complex (hsRHA dsRBD$_2$ and GC10 duplex colored in green and gray, respectively) and NF90-dsRBD$_2$–18mer dsRNA complex (NF90 dsRBD$_2$ and 18mer dsRNA colored in limegreen and gray, respectively). The PDB ID of NF90-dsRBDs-18mer dsRNA complex is 5DV7. The critical residues in regions 1, 2 and 3 required for dsRNA recognition are shown in stick mode. (D) Superposition of dsRBD$_1$ (cyan) and dsRBD$_2$ (magenta) of MLE with Rnt1p dsRBD (orange) (PDB ID: 2LBS). (E) Superposition of dsRBD$_1$ (cyan) and dsRBD$_2$ (magenta) of MLE with dsRBD$_1$ (purple blue) and dsRBD$_2$ (light pink) of TRBP (PDB ID: 5N8M).

Supplementary Figure S4. (A) Representation of the average enrichment (IP/Input) of roX2 and Pka RNAs GFP-tagged MLE$_{FL}$-wildtype and the indicated mutants in RIP assays, showing minimal pka recovery in all samples, as shown in Figure 5B. RNA abundance was quantified in qRT-PCR using the primers provided in Table S2. (B) Representative western blot of roX2 RIP Input fractions. A GFP antibody was used to

detect GFP-tagged MLE_{FL}-wildtype and the indicated mutants. Western blot using an αLamin-specific antibody was performed as loading control. (C) The RNA-binding affinities of MLE $dsRBD_{1+2}$ for R2H1, $R2H1^{\triangle C135\text{-}U140}$ and other regions of roX, determined by FP experiments. $R2H1^{\triangle C135\text{-}U140}$ exhibits the same construct of R2H1 in our crystal structure of MLE $dsRBD_{1+2}$-R2H1 complex. K_d values and the corresponding standard errors were determined as described in the Methods. (D) MALS measurement from SEC of 2 mg/ml MLE $dsRBD_{1+2}$-R2H1 complex at a flow rate of 0.6 ml/min. The MLE $dsRBD_{1+2}$-R2H1 complex eluted as one peak, indicative of an average molar mass of 46.7 kDa, suggesting that the binding stoichiometry of MLE $dsRBD_{1+2}$ and R2H1 in solution was 1:1. Light scattering is shown as a function of elution time (solid line, right axis). Calculated molar mass is shown for the peak (square, left axis).

Supplementary Figure S5. (A) Schematic representation of the *mle* mutagenesis map using precise gene editing based on homologous recombination induced by the CRISPR/Cas9 system. (B) qPCR analysis of genes on X chromosome and autosomes in *wildtype* and $mle^{\Delta RBDs}$ 2nd instar larvae. Error bars are defined as the s.d. of triplicate experiments. The expression level of each gene was normalized to *wildtype* and set to 100% for each gene in *wildtype*. (C) qPCR analysis of MSL-DCC component genes in *wildtype* and $mle^{\Delta RBDs}$ 2nd instar larvae. Error bars are defined as the s.d. of triplicate experiments. The expression level of each gene was normalized to the autosomal gene *Pka*.

Supplementary Table S1. Binding affinities of wild-type and mutant MLE dsRBDs proteins to wild-type and mutant R2H1

Protein	RNA	K_d (μM)	K_{rel}^*
MLE $dsRBD_{1+2}$ (wildtype)	R2H1 (wildtype)	0.20 ± 0.03	1
K4A	wildtype	4.18 ± 0.27	20.9
K4E	wildtype	6.55 ± 0.56	32.8
S5A	wildtype	1.80 ± 0.24	9.0
K4A/S5A	wildtype	4.49 ± 0.54	22.5
K4E/S5A	wildtype	8.05 ± 0.34	40.3
N29A	wildtype	0.43 ± 0.07	2.2
N52A/K53A/K54A	wildtype	7.70 ± 0.36	38.5
K58A	wildtype	2.38 ± 0.13	11.9
N52A/K53E/K54E/K58E	wildtype	12.50 ± 1.51	62.5
K171A	wildtype	4.58 ± 0.68	22.9
K171E	wildtype	8.21 ± 0.26	40.1
E172A	wildtype	0.18 ± 0.04	0.9
N175A	wildtype	1.31 ± 0.26	6.5
K171E/N175A	wildtype	10.34 ± 1.14	51.7
E195A	wildtype	0.15 ± 0.03	0.75
H196A	wildtype	3.02 ± 0.20	15.1
H196E	wildtype	7.24 ± 0.65	36.2
N223A/K224A/K225A	wildtype	18.92 ± 1.45	94.6
K229A	wildtype	0.88 ± 0.18	4.4
N223A/K224E/K225E/K229E	wildtype	60.06 ± 6.55	300.3
wildtype	U118C/A119G	0.42 ± 0.06	2.1
wildtype	U124A	1.03 ± 0.12	5.2
wildtype	U134C/C135G	0.43 ± 0.05	2.2
wildtype	C147G	1.97 ± 0.21	9.9
wildtype	G148U	0.45 ± 0.03	2.3
wildtype	U158G	0.29 ± 0.03	1.5
wildtype	C163U	0.74 ± 0.07	3.7
wildtype	G164A	1.85 ± 0.24	9.3
wildtype	G164U	0.80 ± 0.08	4
wildtype	$R2H1^{\triangle C135-U140}$	0.39 ± 0.06	1.9
wildtype	R1H1	0.34 ± 0.03	1.7
wildtype	R2H2	1.62 ± 0.13	8.1
wildtype	R2H3	58.84 ± 7.02	294.2
MLE $dsRBD_1$	wildtype	8.72 ± 0.83	43.6
MLE $dsRBD_2$	wildtype	0.91 ± 0.10	4.5

K_{rel}^* represents either the affinity of mutant MLE dsRBDs proteins for the wildtype R2H1 or the affinity of wildtype MLE dsRBDs proteins for the mutant R2H1 relative to the affinity of wildtype MLE dsRBDs for wildtype R2H1.

Supplementary table S2. Primers used for qRT-PCR analysis.

Primer name	Primer orientation	Primer sequence	group
roX1	Forward	ATGCGAGCGAGACAATGATACT	MSL-DCC complex-related
	Reverse	GACTTGCAGTCCGCCCTATG	
roX2	Forward	AGCTCGGATGCCATCGAAA	
	Reverse	CGTTACTCTTGCTTGATTTTGCTTCG	
mle	Forward	CGACCAGGCTTCTGCTTCA	
	Reverse	CCGGCGTAAGATTGTCCTCTAG	
msl2	Forward	GCAAGTTGAGGAATCTGATG	
	Reverse	GTTTGTGTAGGTGACTGTGAAG	
mof	Forward	ACGTCCACTATGTTGGTCTCAATC	
	Reverse	CGCATTGTCCGAGATCCTATG	
6-Pgd	Forward	TGCTGGTCAAGGCTGGAAGT	X chromosome -linked
	Reverse	GAGATGTGTCCTGATACTCCGAGTT	
BR-C	Forward	CTCAAGAGCACACCCTGCAA	
	Reverse	CGTGCAGGTCCATGAAGTTG	
CKIIβ	Forward	CCTGGTTCTGTGGACTTCGT	
	Reverse	GTAGTCCTCATCCACCTCGC	
GstT3	Forward	GACATCTTTGCAGCCTGTGA	
	Reverse	GCCCTGATCTTGGGGTACTT	
Pka	Forward	TTCTCGGAGCCGCACTCGCGCTTCTAC	Autosomal euchromatic
	Reverse	CAATCAGCAGATTCTCCGGCT	
Dmn	Forward	GACAAGTTGAGCCGCCTTAC	
	Reverse	CTTGGTGCTTAGATGACGCA	
RpL32	Forward	GCTAAGCTGTCGCACAAATG	
	Reverse	ACGTTGTGCACCAGGAACTT	
GAPDH	Forward	GCCCTGAACGGCAAGCT	
	Reverse	GTAAGATCCACAACGGAGACATTG	
ary	Forward	GTACAAGCGCTCTGCAAGTC	Y chromosome -linked
	Reverse	ACGTAGTAACCGAGTATCCGC	
